# Transforming Life: A Broad View of the Developmental Origins of Health and Disease Concept from an Ecological Justice Perspective

**DOI:** 10.3390/ijerph13111075

**Published:** 2016-11-03

**Authors:** Susan L. Prescott, Alan C. Logan

**Affiliations:** 1 International Inflammation (in-FLAME) Network, Worldwide Universities Network (WUN), 35 Stirling Hwy, Crawley 6009, Australia; aclnd@cfs-fm.org; 2 School of Paediatrics and Child Health Research, University of Western Australia, P.O. Box D184, Princess Margaret Hospital, Perth 6001, Australia; 3 PathLight Synergy, 23679 Calabassas Road, Suite 542, Calabassas, CA 91302, USA

**Keywords:** urbanization, socioeconomic, disadvantage, green space, environmental justice, depression, empathy, marketing, biodiversity, microbiota

## Abstract

The influential scientist Rene J. Dubos (1901–1982) conducted groundbreaking studies concerning early-life environmental exposures (e.g., diet, social interactions, commensal microbiota, housing conditions) and adult disease. However, Dubos looked beyond the scientific focus on disease, arguing that “*mere survival is not enough*”. He defined mental health as fulfilling human potential, and expressed concerns about urbanization occurring in tandem with disappearing access to natural environments (and elements found within them); thus modernity could interfere with health via “missing exposures”. With the advantage of emerging research involving green space, the microbiome, biodiversity and positive psychology, we discuss ecological justice in the dysbiosphere and the forces—financial inequity, voids in public policy, marketing and otherwise—that interfere with the fundamental rights of children to thrive in a healthy urban ecosystem and learn respect for the natural environment. We emphasize *health* within the developmental origins of health and disease (DOHaD) rubric and suggest that greater focus on positive exposures might uncover mechanisms of resiliency that contribute to maximizing human potential. We will entrain our perspective to socioeconomic disadvantage in developed nations and what we have described as “grey space”; this is a mental as much as a physical environment, a space that serves to insidiously reinforce unhealthy behavior, compromise positive psychological outlook and, ultimately, trans-generational health. It is a dwelling place that cannot be fixed with encephalobiotics or the drug-class known as psychobiotics.

## 1. Introduction: Mere Survival Is Not Enough

“*But the quality of the environment cannot be measured only in terms of gross defects such as air, water, or food pollution. Environmental conditions experienced early in life (including the formative months before birth) cause the most profound and lasting changes in man...the maintenance of biological and mental health requires that technological societies provide in some form the biological freedom enjoyed by our Paleolithic ancestors.*”

Rene Dubos, 1970 [1]

Renowned microbiologist and environmentalist Rene Dubos was a pioneer in the “early origins” concept of health and disease. Throughout the 1960s he and colleagues published multiple studies demonstrating the long-lasting influences of maternal stress, nutritional deficiencies and gut microbial alterations on offspring health within various rodent models. In addition to his classic 1966 paper “Biological Freudianism: lasting effects of early environmental influences” (reprinted in his honor within a more recent issue of the *International Journal of Epidemiology*) [2], much of this “early origins” bench work is summarized in his book *Man Adapting* [3].

There is little doubt that Dubos and his colleagues paved mechanistic roads to the contemporary developmental origins of health and disease (DOHaD) concept. Although Dubos connected detrimental early-life environmental influences to subsequent *disease*, he was *equally* concerned with the ways in which the progressive *loss* of (evolutionary-rooted) environmental exposures could erode health *in absentia*. Included in more passive or less obvious aspects of what might be “missing” was diminished exposure to natural environments, including unseen aspects such as microbes. He proposed that in humans, socioeconomic status was a likely determinant of the “*intensity of the inflammatory response to the so-called ‘normal’ flora*” in the context of dietary practices, commensal microbiota and food utilization [3].

## 2. Roadmap to the Current Review

Here, we expand upon the arguments of Dubos in the context of DOHaD, urbanicity and socioeconomic disadvantage. While the absence of disease allows us to survive, we argue that preventing disease is not the ultimate goal of DOHaD. We emphasize the word *health* within the DOHaD rubric and suggest that greater focus on positive (beneficial) chemical and non-chemical, biotic and abiotic, family, neighborhood and societal “exposures” might uncover mechanisms of resiliency that go far beyond disease prevention and extend into health. What’s more, we propose that layering positive “exposures” during all critical developmental stages will have a synergistic effect such that the sum of positive pathways to health might be more than that of their individual parts.

In our perspective review, we build upon the speculations of Dubos with the advantage of rapidly-evolving research exploring the role of positive psychology, biodiversity and natural environments on human health, and, of course, the microbiome revolution that continues to underscore the critical role of early-life microbial exposure in long-term health. We highlight the emerging research on *positive emotions* (rather than exclusively negative emotions typical of the study of depression and/or anxiety) and their associations with immune function, stress physiology and non-communicable disease (NCD) risk [4,5,6]. Further, we explore the rapidly-evolving and no less interconnected realm of nutrition and mental health [7].

Accepting the fundamental truth that there is no health without mental health (defined as the ability to fulfill human potential), we use the vantage of mental health to allow for a vision of equitable urban environments. We will attempt to sew together seemingly disconnected threads of research to make our argument that the interconnectivity of life (biodiversity) and lifestyle (bios), cannot be separated from the causes and consequences of the social policies and practices that either promote or prevent complete mental health. The entire discussion tales place in the context of attaining fundamental rights to achieve one’s potential; equity of the ecosystem.

## 3. Ecological Justice as a Basic Human Right

In referring to ecology, and our later discussion of “ecological justice”, we do so from a perspective in which ecology (“eco” from Greek roots *οἶκος* or *oikos*; house/dwelling place; hence study of the dwelling place) encompasses all lines between internal ecosystems, a single city residence occupied by an individual, through community neighborhoods, and on to surrounding municipal and national dwellings (which house the houses), and outward to our large, universally shared *n* = 1 residence of Earth. Put simply, the way in which microbes sit on a single intestinal villus might contribute to health is not unrelated to the larger ecosystems that impact its human host. For example, there is much talk of the microbiome, but little discussion of the ways in which the policies and practices in the dwelling place of corporate boardrooms (e.g., profits), or the houses of government (e.g., policy or absence of policy concerning corporate practice), insinuate themselves into microbial ecology.

At the outset we frame this ecological vantage by reminding the reader that according to the United Nations, it is a fundamental right of every child to be supported in the development of “*talents and mental and physical abilities to their fullest potential* [and] *respect for the natural environment*” [8]. Thus, the interconnectedness of life and lifestyle cannot be removed from dialogue and action toward achieving and maintaining fundamental rights of childhood development. Any policy or practice that compromises the fulfillment of potential (i.e., health) and the development of respect for the natural environment (i.e., biodiversity) is therefore at direct odds with childhood rights. From our perspective, the microbiome revolution has opened a Pandora’s Box for any remaining attempts to place sole responsibility on individuals or communities for the lifestyles that compromise life.

## 4. Defining Grey Space and the Green Distinction

**Figure 1:**
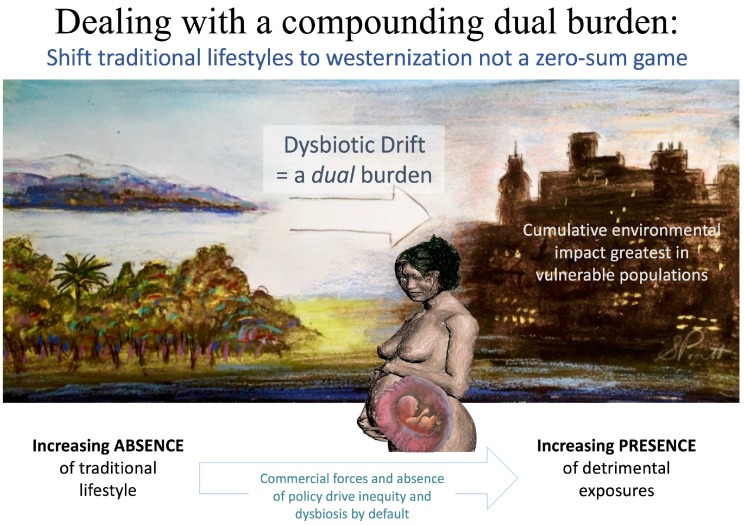
Dysbiosis (defined as “life in distress“) is the result of the modern environmental changes that are adversely affecting all ecosystems, progressively displacing “green space“ with “grey space“ and creating conditions that erode physical, mental and societal health from the first moments of life. This dual burden is compounded and exacerbated by the greyspace environment that perpetuates unhealthy behaviour, with lifelong effects. Commercial forces and absence of policy drive inequity and dysbiosis by default.

We entrain our DOHaD perspective to what we have previously described as urban “grey space“, areas that may include disproportionately higher industrial operations, commercial activity and major transportation routes, with resultant noise stress and excess light at night (LAN). Residential proximity to higher levels of grey space and less equitable access to biodiversity (a product of so-called “green” vegetation-rich and “blue” waterside areas) are an often-overlapping burden in disadvantaged populations (Figure 1). However, our definition of grey space is not based on specific units of analysis, single indicators and/or jargon related to quantitative toxin-based environmental impact assessments. These can obscure the ability to understand the sum of the total reality endured by the socioeconomic status SES disadvantaged [9].

**Figure 2:**
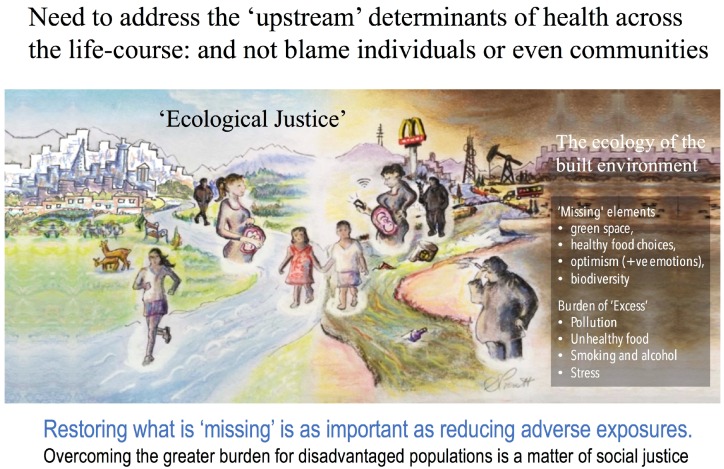
The Tale of Two Cities: Addressing the “upstream“ drivers of health and human potential. While individuals can influence their environment, the environmental effects on individuals (and their opportunities and choices) are much greater. These operate widely to influence both the health of environments and the health of individuals across life.

The disproportionately higher presence of bars, liquor stores, convenience stores, fast-food outlets, and tobacco vendors in disadvantaged areas *help* define the grey aspects of place; however, it is more than mere structural presence. Toxin and remote sensing indicators cannot capture the profit-driven marketing, billboards, sidewalk signage, in-store magnification of unhealthy products and targeted screen media delivery that make grey space an entirely different “mental” environment (Figure 2). As we will argue, these aspects of grey space serve to insidiously reinforce unhealthy behavior, compromise positive psychological outlook and, ultimately, trans-generational health.

Importantly, in our later discussions of green space, natural environments and the potential health benefits of maintaining psychological connections to nature (i.e., nature relatedness), we underscore that ours is not an attempt to privilege or romanticize a notion of a “pure“, “green“ way of living that sits in a neat mirror image of grey space and urbanicity. Undoubtedly, the urban built environment, mixed with green and grey as it may be, can promote or detract from health in ways that are predominated by socioeconomic factors. Ours is not necessarily a “back to nature“ call. However, if we can succeed in our attempts to demonstrate that a more equitable distribution of access to quality natural environments, fresh healthy, minimally-processed foods and the “assets“ of positive psychology are part of ecological justice—capable of narrowing the SES health gap—then perhaps ours is a “forward to nature“ message.

## 5. Broadening Developmental Origins of Disease Paradigms

DOHaD research has centered upon three potentially-overlapping early-life environmental influences that have been linked to subsequent disease: nutrition (that is, extremes of undernutrition or, conversely, overnutrition via energy dense-nutrient-poor dietary patterns), stress (including social adversity), and the burden of environmental toxicants [10,11,12]. These exposures can act alone or in concert during specific windows of developmental plasticity. Allostatic load (wear-and-tear, cellular damage of physiological dysregulation; e.g., sustained elevations in stress hormones and/or inflammatory immune chemicals) and epigenetic mechanisms (e.g., methylation of the CpG dinucleotide in DNA, acetylation and methylation of histones, and the binding of small non-coding RNAs to DNA) can manifest as disease and dysfunction over the life course.

There has been less focus on the upstream ecological drivers of DOHaD mechanistic pathways. That is, politics and policies. Less discussion of the impact of missing exposures (e.g., biodiversity (microbial and macroecological) and positive emotions discussed later). The focus on “preventing disease“ has obscured the more expansive goal of “promoting health“ in the broadest sense of achieving human potential.

Notwithstanding the importance of specific developmental windows, in our view there are no specific life-course timeframes that determine where developmental “origins“ begin and end; paternal stress may influence sperm microRNA which, in turn, could influence offspring depression and anxiety [13]. Overall, trans-generational research has made it clear that ancestral trauma and stress are relevant to where the line of origin might begin [14,15]. DOHaD is a discussion for all.

## 6. Mental Health, Societal Health: Avoiding Mother Blame

There has been a consistent reporting of increased psychological distress in most (8/11) global surveys over the last several decades [16]. While global “epidemics” of *diagnosable* major depressive disorders (MDD) and anxiety disorders remain a matter of debate, collectively these data indicate that psychological distress is at unacceptably high levels among adults and youth in westernized nations [17,18]. Moreover, increasing perceptions of stress with modernity may be manifesting as somatic complaints [19,20].

Although 1/3 of adults presenting to primary care have symptoms of depression, anxiety, and/or alcohol problems, only a minority (<8%) articulate these overtly as their primary complaint [21]. Subthreshold anxiety and depression are increasingly common in youth and represent a significant public health concern in developed nations [22,23,24], often matching the levels of psychological distress and diminished perceptions of health as defined disorders [25,26]. Importantly, there are noteworthy global trends of a consistent generational drift away from empathic perspective taking and toward narcissism—relevant to our discussion on empathy and positive psychology at some length later [27].

In the developmental context, maternal depression and anxiety in pregnancy have wide ranging implications for the long term burden of disease of the next generation, and for society. For example, emotional distress may provoke unhealthy dietary patterns [28] and higher risk of preterm birth and low birth weight [29,30], which are in turn associated with higher risk for both clinical and subsyndromal mental disorders in offspring [31,32,33,34,35,36,37,38,39,40,41,42]. Importantly, subthreshold forms of depression can be identified in preschool children and represent a robust marker of subsequent clinical depression [43]. Youth with subthreshold symptoms are on a higher risk trajectory toward both adult mental health disorders [44] and other non-communicable disease (NCD) with associated co-morbidity and diminished life quality later in life [45,46].

Anticipating that additional investigation will only heighten the already existing mass of research linking maternal/paternal depressive symptoms and lifestyle with offspring weight gain, early-life affect/emotional regulation and altered parent-child dynamics [47,48], we must surely ask what ecological factors drive depression or, conversely, positive emotions in the first place? Upstream discourse is necessary because communication surrounding DOHaD runs the risk of holding an individual woman as the sole proprietor of responsibility (and therefore, subject of “mother-blame”) concerning fetal and early-life health [49]. Moreover, this risk in communication can extended to inferences that entire disadvantaged communities are not doing enough—i.e., “community blame” [50]; meanwhile, the more powerful forces—e.g., profits, elitism, neoliberal ideology—[51,52,53] that actually push against individual and community-level lifestyle (e.g., diet) are left out of the discourse.

Society at large (its policies and bio-eco-psychological influences [54]) contribute to the nurturing of each individual fetus, infant, child and adolescent. It does so vis-à-vis its ability to provide a supportive environment for pre-conception health, maternal and paternal health, and that of adult caregivers of any age. It does so by supporting the community in its efforts in providing the best possible “neighborhood womb“ (Figure 2). Although there may be critical developmental windows, privileging DOHaD communication to narrow time frames may also run the risk of inferring that societal policies and practices (those facilitating the presence of chemical/non-chemical toxins/stress and absence of positive exposures) are acceptable once a child is 3, or that they are of less relevance through youth development and for men and women planning to have a child.

## 7. The Prism of Socioeconomic Gradient

Not only are mental disorders significantly more common in urban regions of Western nations [55,56], the also they sit on an SES gradient [57,58] with depression and fatigue particularly associated with social disadvantage [59]. Disparities in mental health are also evident in SES disadvantaged children and youth [60], most striking in developed nations where inequities are greatest [61]. Mental disorders can contribute further socioeconomic hardship [62] and reduce resiliency. In a large Canadian community study, full recovery from depression was predicted by being Caucasian, affluent and without a significant history of childhood adversities [63]. On the other hand, positive emotions (e.g., optimism and others to be discussed in detail later) are emerging as assets of resiliency that are consistently linked with socioeconomic advantage [4].

Preterm delivery and suboptimal birth weight are also more likely to occur in disadvantaged populations [64,65]. In turn, even moderate preterm (32–36 weeks) birth multiplies the independent risk of low SES on behavioral and emotional problems in children [66]. Thus, supporting healthy pregnancy outcomes represents a salient and early way to begin to dismantle inequalities and transform life.

DOHaD as a collective group may be undervaluing its role in taking some of the valuable fruits of its labor—mechanistic science—toward ecological justice and public health advocacy. Moreover, in the hunt for mechanisms of disease, it may overlook broad social science research. For example, given known connections between parental mental health and offspring health, emerging research connecting relatively small wage increases with subsequent reductions in depression (in SES disadvantaged workers) [67,68] should be of high-level interest to DOHaD. Lack of a living wage magnifies the attraction of unhealthy lifestyle (e.g., dietary choices) and alters opportunity to spend time in natural environments (e.g., time constraints of working additional hours or multiple jobs), even if such areas were equitably distributed.

## 8. Lifestyle Factors: The Vicious Cycle of Ill-Health

Accumulating evidence suggests that modernity and rapid global urbanization (and more specifically, many of its policies and practices described later in the “Grey Space” section) is encouraging a lifestyle at odds with our evolutionary past. For the nearly 3-million-year history of our genus we have maintained a physically active lifestyle, consumed minimally processed foods and lived entrained to the light-dark cycles. The links between this ancestrally-discordant lifestyle and most modern NCDs is apparent [69]; thus, to some extent, increasingly prevalent NCDs may be described as “ancestral mismatch disorders“.

From the mental health perspective, nutrition is now proven to be an essential consideration in short and long-term neuro-emotional health, particularly in positive mental outlook [7,70]. At the population level, adherence to dietary patterns with relatively lower amounts of highly-processed foods (i.e., traditional patterns known to be less inflammatory) is associated with lowered risk of depressive symptoms of anxiety and depression [71,72]. From the DOHaD perspective, perinatal dietary quality has been linked to good mental health in offspring, and early life nutritional quality with academic performance [73,74,75].

The palatability of the engineered, ultra-processed Western diet is high, an attribute that may temporarily lower stress and improve mood [76]. Thus, this dietary pattern may be reinforced through “self-medication“ [77]. In experimental studies, withdrawal from this palatable, western diet leads to notable changes in gene expression governing stress physiology [76]. Simply put, weaning off such diets appear to be stressful; research on the stress-attenuating effects of sucrose [78] and mood-lifting effects of fat [79] supports this notion in humans. Moreover, it is easy to imagine how difficult this might be when the efforts are hampered by contextual psychosocial stress and a “grey space“ environment that cannot be fully replicated in rodent studies.

Relevant to our later discussions specific to SES disadvantage, research shows that humans often increase their consumption of calorie-dense, nutritionally-poor “comfort foods“ when confronted with psychological stress [80]. In a vicious cycle, the draw towards unhealthy foods is associated with chronic depressive symptoms and psychological distress. Like many lifestyle variables, nutritional quality is intertwined with sleep; low fiber and high saturated fat and sugar intake is associated with lighter, less restorative sleep with more arousals [81].

Adequate and appropriately timed sleep is essential to health and quality of life throughout the life course. Sleep disturbances have been linked to increased risks of cesarean delivery and preterm birth [82]. Sleep, circadian and biological rhythm disruptions are linked to depressive symptoms during and after pregnancy [83,84]. Antenatal paternal depression has also been noted in association with sleep problems [85]. Parental depression has, in turn, been associated with disturbances in infant sleep patterns [86]. Adequate sleep during childhood is emerging as a critical determinant of resiliency (i.e., achieving WHO *health*) and diminished risk of behavioral problems [87,88].

Screen time has been linked to obesity and mental health problems in children and adolescents. These associations may carry forward into adulthood [89,90]. Minimizing screen time and increasing outdoor play time in very young children may have overlapping benefit on sleep quality [91]. From an academics perspective, higher levels of screen time are associated with lower academic achievement [92]. In addition, greater dependence upon texting and media use has been associated with delay discounting (discussed below) [93]. Screen time as it relates to diminished psychological well-being clearly interacts with physical activity, sleep quality, and social support [94,95], yet it may also be an independent variable in the risk of depression [96].

## 9. Disadvantage and the Cognitive Tax

Neighborhood-level disadvantage is associated with NCDs and an increased likelihood of experiencing poor health at a much younger age over the life course [97,98]. Of particular importance to DOHaD, lower perceived neighborhood quality (reported by minority women in metro Detroit, USA) is associated with higher depressive symptoms and stress during pregnancy [99]. Perception matters: perceived neighborhood walkability is associated with far higher weekly physical activity and higher perceived availability of healthy foods (vs. lowest) is associated with almost 50% higher intakes of fruits and vegetables [100].

Although convenient (and conveniently common) to consider individual self-control as a primary pathway to NCD reduction and developmental disease resiliency via adherence to healthy lifestyle, the environmental forces pushing down on the bar of self-control (e.g., advertizing and others to be discussed) can be unyielding [53,101]. In addition to the direct influences of neighborhood grey space and marketing forces that push unhealthy lifestyles by default, it is worth noting that cognitive and motivational barriers to individual behavioral change can be a product of both disadvantage itself (e.g., poverty) and the NCDs that are highly associated with disadvantage (e.g., obesity or depression).

First, poverty-based concerns are an omnipresent tax on finite mental resources [102]. This has been demonstrated in laboratory and field studies conducted in North America and India. Focus on monetary concerns appears to diminish cognitive resources that could otherwise be directed to other problems or maintenance of healthy lifestyle habits. In terms of effect size, the deficit of cognitive resources induced by poverty-associated financial concerns is akin to that found when sleep researchers deprive subjects of a full night of sleep or a loss of 13 intelligence quotient IQ points [103].

Second, it is easy to visualize how societal pressures and the neighborhood-level grey space environment might contribute to feelings of powerlessness and impaired optimism. Experimental induction of merely *feeling* poor has been shown to increase caloric consumption [104]. Further, when researchers set up a game of with a rigged social outcome (i.e., subjects induced to feel of low social status with resultant decreased feelings of pride and powerfulness) young minority participants consume significantly more calories and a higher proportion of their daily calorie needs (in the ad libitum buffet meal vs. subjects manipulated to high social status) [105].

In sedentary adults the motivation to engage in physical activity is low, and the normal post-exercise lift in mood is often not experienced. For example, in those with NCDs such as type-2 diabetes, depression and/or obesity (vs. healthy/normal weight controls), motivation is a primary barrier to physical activity [106]. The same cognitive load carried by disadvantaged populations may also impair motivation to exercise simply by influencing motivation. Mental fatigue primed prior to or during exercise via cognitively demanding tasks can increase perceived exhaustion during and after exercise [107,108].

The line between initiation (short-term adoption) and long-term adherence of voluntary physical activity appears to be drawn by *identified* behavioral regulation (e.g., consideration of potential beneficial outcomes to self) and *intrinsic* motivations (e.g., inherent enjoyment of the activity) [109,110]. Thus, consistent cognitive demands, delay discounting, anticipation of fatigue/exhaustion and low levels of exercise enjoyment can compromise engagement in routine physical activity. Moreover, sleep deprivation and circadian disruptions—another burden carried by the disadvantaged—can also have negative influences on physical and mental performance during exercise [111].

Studies demonstrate that simple awareness of the benefits of exercise may not be enough for patients with NCDs; perceived exertion is higher, pleasure ratings and energy levels lower [112,113,114]. This, or course, impairs motivation to engage in subsequent exercise. On the other hand, when positive emotions are in tandem with the experience of exercise and recovery, future participation is more likely [115,116].

## 10. Lifestyle and Delay (Temporal) Discounting: Future Rewards?

Research pertaining to delay (also called temporal) discounting may be one of the most oft-overlooked topics in chronic NCD and DOHaD discussions; briefly, delay discounting refers to the willingness to postpone (delay) immediate rewards in favor of potentially larger benefits in the future. Discounting the potential value of future rewards, and instead prioritizing smaller immediate rewards, is referred to as steep discounting. Steep discounting is often code for diminished consideration of future consequences and impulsivity, both of which are linked with depression, obesity and unhealthy lifestyle choices [117,118]. For example, higher concern for future consequences is associated with preventive care, optimism, positive affect, lower aggression, interpersonal wellbeing and higher life satisfaction [119].

Steeper gradient of discounting future rewards is also associated with socioeconomic disadvantage [120,121,122]. Cognitive load, stress and diminished mental outlook and even (those outside conscious awareness) can magnify delay discounting [123,124]. Of importance to our grey space theme, physical aspects of the built environment such as fast food outlets may increase delay discounting in stealth ways. For example, discounting the value of a future financial reward and opting for smaller immediate gains is more likely while answering questions in the vicinity of a fast-food outlet (vs. other food establishments). Moreover, in neighborhoods with higher concentrations of fast food outlets, respondents are also more likely to opt for smaller immediate rewards, wave off larger future gains and have diminished levels of savoring (i.e., ability to take notice of positive experiences, an asset linked to mental well-being) [125,126].

On the other hand, images of (or actually being in) vegetation-dense green space have been shown to curb delay discounting in several studies [127,128,129]. It is also encouraging that even subtle reminders of prospect—simple outlooks toward the future via prospective imagery and stimulation of general future experiences—can help to level the discounting gradient [130,131]. Sleep quality appears to be essential for broad consideration of future consequences. It helps keep impulsivity in check and allows an individual to envision the efforts required to obtain future rewards as being less burdensome [132,133,134,135].

## 11. Positive Emotions and States: Neglected Discussion

While there has been much focus on the detrimental effects of maternal/early-life negative emotions and states, the independent links between positive emotions and health has largely escaped discourse. Positive and negative emotions are not opposite and are only correlated at a modest level [136]. Positive affect has been associated with good sleep [137] and healthy pregnancy outcomes [138]. In 28-year prospective research, infant positive affect (parent rated at baseline) uniquely predicts adult life satisfaction, workplace hope and optimism [139]. Early adolescent positive affect also predicts healthy adult relationships, workplace competency and self-worth [140].

Positive emotions such as awe are linked with lower inflammatory immune markers such as interleukin-6 (IL-6) [141]. Happiness, as distinct from negative emotions such as depression/anxiety, is associated with lower intercellular adhesion molecule-1 (ICAM-1) promoter methylation [142]. ICAM-1 is associated with progression of depressive symptoms, cognitive decline and cardiovascular disease [143,144]. Research shows that the frequency of daily positive events is associated with lower IL-6 and C-reactive protein [145]. In a study examining the emotional responses to daily stressors, elevated IL-6 was associated with decreased positive affect [146].

Optimism, or possessing positive outcome expectancy for future events across life domains, appears to be a particularly important asset within positive psychology. Higher levels of optimism have been associated with lower inflammatory cytokine levels and C-reactive protein [147], and lower inflammatory response to experimental stress [148]. However, optimism is an asset highly associated with socioeconomic advantage [149] and healthy lifestyle habits [5,150,151]. Optimism also has complex ties to sleep; optimism improves sleep, yet poor sleep appears to promote pessimism [152].

Purpose in life, increasingly connected to NCD risk reduction and health and well-being [153,154], is also an important factor that warrants attention. Generally, a high degree of purpose in life is associated with goals, meaning, and a long view. In community samples, high scores on purpose in life are associated with lower (shallow) discounting rates. Thus, a broader life view may diminish impulsivity [155]. Matching this, prospective research shows that higher levels of purpose in life predicts lower allostatic load [156] and purpose in life predicts greater use of preventive healthcare measures [157]. Research shows that purpose in life can mediate relationships between neighborhood disorder and parenting stress in disadvantaged communities [158]; thus, DOHaD conversations around purpose in life may be particularly important.

Empathy is the ability understand or make accurate inferences based on the experiences of another; the combined cognitive and emotional aspects of empathy allow one to take the perspective of another, and to experience some of their emotions in a vicarious way. It has been linked to life satisfaction, wellbeing, rich social networks, healthy relationships and workplace performance, accommodative behavior, and pro-social activity [159,160,161]. Prospective research indicates that higher levels of early-life empathy predict social competencies [162]. In youth, higher empathic concern is linked to greater connectedness to nature; thus, being able to take the point of view of another person and perceiving the natural world as important to health and wellbeing are intertwined [163]. Among youth, elevations in empathic concern over time have been associated with decreases in pro-inflammatory cytokines [164].

## 12. Natural Environments, Missing Exposures Exaggerate Disparity

“*Urban dwellers never have the chance to see the Milky Way, or a night radiant with stars, or even a truly blue sky. They never experience the subtle fragrances peculiar to each season; they lose the exhilaration of early spring and the delightful melancholy of autumn. The loss of these experiences is more than an aesthetic affliction; it corresponds to a deprivation of needs which are essential to physical and mental sanity, because they were indelibly woven in man’s fabric during his evolutionary past.*”

[165]

Natural environments are areas typically defined as those that are *relatively* unchanged or undisturbed by human culture, although they can include areas that are designed, manipulated and sustained by human interventions [166]. In the context of urban settings this may include gardens, parks, forests and waterside areas. Natural environments as whole (and specific elements within them: e.g., airborne phytoncides released from plants, natural light, negative ions, sounds, tactile opportunity, microbes (discussed in detail later)) may buffer stress, improve cognition, facilitate physical activity, encourage social cohesion, and promote overall health and mental well-being [167,168]. Residential proximity to higher concentrations of green space has been linked to lower mortality [169], and this appears to be especially true for disadvantaged communities [170].

**Figure 3:**
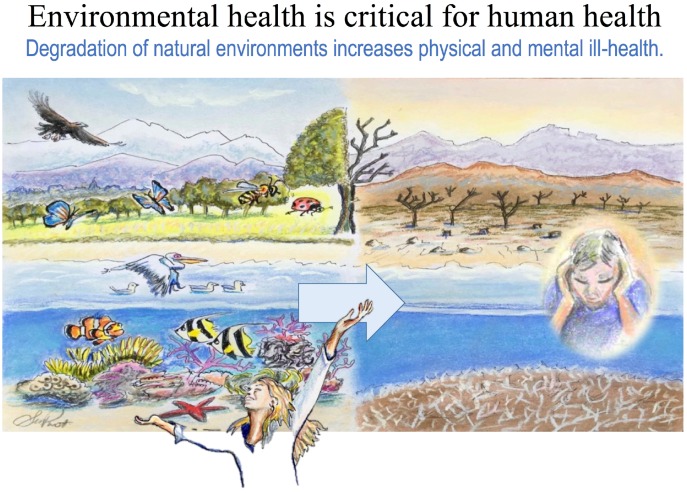
The health of humanity depends on the health of our environment. The physical and mental health consequences of environmental degradation should not be underestimated.

From a developmental perspective, it is noteworthy that residential closeness to green space has been linked with healthy pregnancy outcomes (normal term and/or healthy birth weight) in multiple epidemiological studies [171,172,173,174,175,176,177,178,179,180]. These protective links are intertwined with SES but may be strongest in disadvantaged populations [181]. Surrounding greenness during pregnancy is associated with lower rates of incident asthma in children followed for 10 years [182]. Moreover, green space has been linked to good mental health during pregnancy; the influence of green space on resiliency against depression appears most pronounced in disadvantaged populations [183] (Figure 3).

Higher levels of green space at the neighborhood level have been associated health-related quality of life and reduced aggression in youth, as well as lower risks of depression and/or anxiety in the broad population [184,185,186,187,188,189,190,191]. Research tracking three years of mobility within the U.K. shows that individuals who move into areas with a higher greenness (vs. their previous residence) experience improved mental health [192]. Again, to what extent is this mobility research also capturing exit and entry from/to grey space?

However, unlike the gradient of depression risk that points toward the disadvantaged, the concentration of urban green space, parks, open play space (or perceived and real safety, accessibility, maintenance, quality) and local biodiversity is often slanted in the opposite direction, favoring the affluent and less vulnerable [193,194,195]. Among preschool children, SES and non-accessibility of green space are both associated with mental health problems [196]. Research shows that parks in high-income areas are used more frequently; in part, this may be due to the availability of structured/supervised activities and better marketing/outreach efforts that encourage use [197].

Natural environments research overlaps with screen time. Children residing in urban environments [198] and disadvantaged neighborhoods [199] may have higher daily screen time than rural or affluent counterparts. Research shows that in neighborhoods where walkability is less than optimal, screen time is higher [200,201,202]. When both parents and children perceive their neighborhoods to be safe, physical activity is higher and screen time is significantly lower [203]. Adding to the complexity of oft-isolated research, among children from low-income families in the United States, high levels of screen time are coincident with frequent fast-food consumption [204].

The mental health value of green space as a means to help narrow SES health inequalities has been examined in large-scale epidemiological research throughout European cities. Notably, socioeconomic inequality in mental well-being was 40% (8.1 points on the 5-item World Health Organization Well-Being Index score) narrower among respondents reporting good access to green/recreational areas, compared with those with poorer access [205]. Thus, providing equitable access to natural environments in urban settings can assist in disrupting the usual conversion of socioeconomic inequality to health inequality.

Emerging research shows that the health benefits of visiting natural environments may depend on dose; for example, dose-response analysis indicates that visits to outdoor green spaces of 30 min or more during the course of a week could significantly reduce depression and hypertension within urban populations [206]. Spending time in natural environments has been associated with lower markers of inflammation, stress physiology and oxidative stress [207,208,209]. Residential proximity to and/or engaging with natural environments has been associated with lower cortisol levels [210,211,212,213]. Comparing Scottish socioeconomically disadvantaged neighborhoods, some of which can vary in their green space land use from as low as 14% to as high as 75%, researchers find higher percentages of green space are linked with healthier daytime salivary cortisol patterns and lower perceived stress [214,215].

Access to neighborhood green space has also been linked to healthy sleep [216,217] and a 17-min lunchtime walk in nature (vs. built environment) has been shown to have positive influences on parasympathetic activity during night-time sleep [218]. Normal delivery of adequate light during the day and withdrawal from light at night (LAN) represents an essential biophysical cue during pregnancy. Alterations in the photoperiod (known as chronodisruption) and can interfere with healthy, term pregnancies and have long-term effects on normal offspring [219,220,221]. Artificial LAN has been linked to obesity at the international level [222]. Thus, walkable neighborhoods, quality green space and opportunity for outdoor activity, coincident with lower evening screen time, is connected to sleep hygiene.

In academic settings, proximity to natural environments and classroom views to green space have been associated with positive cognitive development, attention, resilience in stress physiology, and academic performance [223,224,225,226,227]. These results hold for standardized testing even when SES is controlled [223,224]. Moreover, the influence of being in nature or exposure to scenes of nature extends to altruism, helping behavior and social value orientation [228,229,230]. These studies examining cognitive and psychological outcomes lend support to epidemiological findings concerning mental health.

## 13. Nature Relatedness

Individual psychological constructs such as nature connectivity, nature connectedness and nature relatedness (NR) can measure an individual’s awareness, understanding and fascination with the natural world, as well as an interest in and desire for nature contact. Higher scores on these scales have been consistently linked to mental well-being [231,232]. NR is a strong predictor of visitation to local green space and meeting physical activity guidelines within green space [233].

However, researchers must now explore the ways in which NR is developed, perhaps in early life, and to what extent it is influenced by SES, if only through access and experience in nature. If natural environments can reduce health disparities (especially mental health) along SES lines, it would seem valuable to know if NR is a variable in that equation. Indeed, NR may be of relevance to discussion of the microbiome if it facilitates increased contact with diverse microorganisms.

## 14. Humans, Microbes, Environment as an Ecological Unit

“*Thus, life in the world of nature, implying as it does endless contact with all kinds of microbes, early brings forth in animals an adaptive response*”.

[234]

The holobiont view of human life underscores that we are assemblages of different species—persistent symbionts—that make up an ecological unit. The human host and its microbiome (trillions of microbes and their collective genomes) are therefore an ecological community. In turn, as we will discuss in more detail later, the holobiont operates within its own ecological theater—that is, the often inequitable biotic and abiotic neighborhood.

Dating back to the pioneer studies of Dubos, the importance of microbes in early life has been a staple of DOHaD research [235]. Functions of the microbiome include, but are not limited to, “education“ of the immune system, protection against pathogens, maintenance of barriers to the external environment, nutrient production and extraction, increasing bioavailability of dietary phytochemicals, lipid metabolism, provision of short chain fatty acids, production of bioactive metabolites, and detoxification of environmental toxins. Collectively these benefits can be described as ecosystem services [54]. Recognition of these functional attributes often surface as a result of observing the consequences of microbial disturbances via antibiotics, stress, westernized diets and/or lifestyle.

Dysbiosis is a term that translates as “difficult living“ or “life in distress“; given neighborhood grey space, massive health disparities, matters of economic and environmental injustices, biodiversity losses, climate change, rapid urbanization and other threats to ecosystems, it has been argued that dysbiosis can apply at the individual, neighborhood and global level [54,236]. In the context of microbiology, dysbiosis refers to perturbations of at least one, or a combination of the following: loss of beneficial microorganisms, and/or the expansion of potentially harmful microbes, and/or the loss of overall microbial diversity [237].

Emerging evidence suggests that gut microbial dysbiosis may, at least in part, have causal relationships with depressive symptoms, anxiety and mood alterations [238,239,240,241,242]. In experimental research antibiotic exposure during pregnancy disturbs microbiota and causes behavioral deficits in offspring [243], while human studies have linked antibiotic use to depression and anxiety [244]. Pathways of microbial influences on brain development, stress physiology, mood, cognition and behavior include, but are not limited to: immune-mediated pathways, enhancement of nutrient bioavailability and neurotransmitter precursors, support of the gastrointestinal barrier, redox homeostasis, and even direct gut microbe-to-brain communication via the vagus nerve [245]. While most microbiota-mood evidence is confined to rodent studies, there are intriguing human intervention studies suggesting that microbiome therapeutics (e.g., probiotics) can improve short-term mental health and improve quality of life [246,247].

## 15. Encephalobiotics, Promissory Notes

Encephalobiotics are defined here as probiotics, prebiotics, postbiotics, microbes, microbial parts and/or agents that influence the microbiome for cognition, mental well-being and brain health. They are distinct from so-called psychobiotics which are defined as specific living microorganisms used for patients with *psychiatric disorders* [248]. Since psychobiotics are directed at patients with mental disorder, they are by definition tightly-regulated drugs that will require years if not decades of research. That fact hasn’t curbed enthusiasm for hyperbolic media headlines suggesting those with emotional disorders “forget prozac“ in favor of psychobiotics.

Early-life microbial applications may have a place. However, in our opinion, the promise of microbial application (from early life and beyond) for long-term mental health benefit via encephalobiotics will be unrealized if the drivers of dysbiosis (both in its microbial definition and personal, community and planetary “life in distress” definition) remain in place. Neoliberal ideology suggests that dysbiosis at all levels is fixable at the individual level, and in particular, with product application.

The interconnected, patent-driven, biomedical-biotechnology-microbiome zeitgeist has provided plenty of promissory discussions with little mention of SES disadvantage. Thus, discourse concerning the coincident environmental and marketing forces that might cause their need in the first place—i.e., higher need in the very populations least well-equipped to purchase them—is avoided [236]. Absent is the reality that the external built ecosystem (i.e., the neighborhood) maintains a continual undertow toward dysbiosis.

## 16. Hygiene Hypothesis, Dysbiotic Drift

As we illustrate, the drivers of microbial dysbiosis (e.g., westernized/ultra-processed diet, stress, alcohol, tobacco, circadian disruptions, antibiotic use, et al.) are slanted toward the SES disadvantaged in developed nations, and increasingly so in developing nations. The presence of grey space (described in detail later; an environment maintained by marketing forces and absence of policy that could otherwise transform opportunity for health) accompanies disadvantage and contributes to what has been referred to as “dysbiotic drift“ [249]. For example, reduced diversity of colonic microbiota (sampled via mucosal biopsy) is evident among residents of lower socioeconomic status (SES) neighborhoods in North America [250].

Some brief contextual background to illustrate the inequity of ecological medicine may be helpful. Briefly, the hygiene hypothesis and its variants suggested that the global rise in allergic disease is (at least in part) a product of diminished opportunity for early life exposure to diverse microbial exposure via increased hygiene, antibiotics and smaller family sizes and westernized dietary patterns [251,252]. Presently, available evidence supports the hygiene hypothesis; indeed lack of evolutionary-rooted microbial immune priming as an NCD-provocateur may also extend to neurocognitive and mental health [245,253].

One barometer of the hygiene hypothesis, and its links to what might be referred to as “microbial deficit disorders“, includes shifts away from fermented foods that have otherwise been a long-standing part of traditional dietary practices [254]. For example, recent studies show that higher consumption of traditionally fermented foods is associated with reduced risk of atopy in adults [255], lower rates of allergy in children [256], anxiety in young adults [257], and eczema in offspring when consumed during pregnancy [258]. However, in North America, affluent Caucasian women maintaining healthy lifestyles—those at far lower risk of depression—are significantly more likely to consume fermented foods and encephalobiotics [259].

The dysbiotic drift theory of SES disadvantage in developed nations does not hinge on diet alone. Antibiotic exposure is often higher in disadvantaged populations [260,261,262]. The microbes carried by mammals are also a product of the ecosystems in which they reside [263,264,265,266]; given that the Earth is home to upward of 1 trillion microbial species [267], human contact with many of these microbes in natural environments (the ecosystem in which we once spent the majority of our time) may have evolutionary-rooted, health-protective properties.

The SES deprivation of natural environments may be an unrecognized consideration in microbiome and health discussions. For example, in humans the level of green space and biodiversity of vegetation surrounding one’s residence appears to drive diversity of cutaneous microbial ecosystems, including *Gammaproteobacteria* and key species within this microbial family [268,269]. Green spaces contribute unique and diverse beta bacterial signatures to the urban environment [270], and such vegetation makes a significant contribution to the airborne microbial content—up to 10-fold higher than nearby non-vegetated built areas [271].

*Mycobacterium vaccae*, a generally non-pathogenic microbe commonly encountered in natural environments, has been shown to reduce depression and anxiety-like behavior in animals in concert with activation of central serotonergic pathways [272,273]. Additional experimental research shows that heat-inactivated M. vaccae can enhance fear extinction, improve stress coping, and prevent stress-induced dysbiosis [274]. Potential mechanisms include a suppression of pro-inflammatory cytokine production.

Examining the non-dietary and antibiotic pathways to dysbiosis shows that they are essentially the very same roads of exposure travelled by disadvantaged populations. Human and/or experimental research demonstrates that the risk of microbial dysbiosis is increased by acute and cumulative psychological and physical (e.g., thermal, noise) stress [275,276,277,278,279,280,281], environmental pollutants including lead, polycyclic aromatic hydrocarbons and airborne particulate matter [282,283,284], tobacco exposure [285,286,287] and excess alcohol consumption [288]. In addition, the oral periodontopathogen *P. gingivalis*, reported to be much higher in North American disadvantaged populations [289] has recently been shown to cause dysbiosis when swallowed [290]. Other dysbiotic influences which are more common along SES disadvantaged lines include sedentary behavior, circadian disruptions, sleep problems with functional constipation, and low levels of vitamin D [249].

## 17. Grey Space, Inequity and the Environmental Push

Thus far we have presented research highlighting that a preponderance of existing research supports healthy dietary patterns, microbial diversity, and access to quality natural environments as factors in positive mental health. Here, we underscore that research topics such as positive psychology, microbiota, diet and green space research are often placed in silos. Closer examination of the inequity of these elements in SES disadvantaged urban environments reveals their important synergy.

North American parks and their immediate vicinity represent important locations where food and beverages can be purchased (running the gamut from sit-down restaurants with healthy choices to fast-food chains, food carts and vending machines) [291,292]. What’s more, residential proximity to urban green space and greater park access is associated with healthier dietary habits (e.g., more fruits, vegetables, whole grains, nuts/beans, and less fast-food, sodium-rich food and sugar-rich beverages) and lower insulin resistance [293,294,295]. In higher population density areas, relatively more natural food/specialty stores, fewer convenience stores and more physical activity resources are associated with higher diet quality [296]. Lower availability of parks (and open spaces) for physical activity may operate in tandem with greater density of fast-food outlets (and less grocery stores) in the promotion of NCDs, especially for the disadvantaged [297,298].

## 18. Walking in Grey Space

Although it is atypical to invite readers of academic articles to engage in visualization exercises, here we welcome the reader to imagine residing in a socioeconomically deprived community (depicted in aggregate) through the research presented in this section. That is, to fully immerse in the mental environment wherein aggregate individual and neighborhood disadvantage may be classically determined by income, education, social cohesion, racial/ethnic/immigrant segregation, evaluations of neighborhood aesthetic quality, and/or aspects of actual or perceived safety. We recognise the heterogeneity of urban environments in developed nations and that not all disadvantaged areas are alike; however, the visualized community (henceforth referred to as “*our neighborhood*“) may have some, or even all of the aforementioned disparities.

We believe this visualization is important because academic discussions on environment and lifestyle can be reductionist and isolated; visualizing an environment can effectively influence cognition. Our intention is to encourage readers to absorb at least some tiny slivers of the mental environment (described below) that make up the daily reality in SES disadvantaged urban environments. As Dubos stated in 1965 concerning the scientific disconnect:

“*A more disturbing aspect of modern science is that the specialist himself commonly loses contact with the aspect of reality which was his primary concern, whether it was matter, life or man*”.

[299]

We have already highlighted medically-important lifestyle habits that can be coincident with disadvantage. Higher levels of screen time and indoor sedentary behavior, too little or too much sleep (and frequent daytime sleep [300]), dietary patterns of high-calorie, low-nutrient-density foods (including ultra-processed, high sodium, additive-rich foods; and/or less fruits and vegetables), excess alcohol consumption, and tobacco use are characteristic of disadvantage [249]. Some of these habits may extend to “bystanders“ (e.g., second-hand smoke and shared dietary choices) [301].

## 19. Behavioral Reinforcement

To begin, we will find a disproportionate availability of bars, liquor stores, convenience stores, fast-food outlets, and tobacco vendors—which significantly alter the mental landscape of both adults and children [54]. This matters. As much as 31% of the variance in excessive fast-food consumption may be attributable to simply living in urban areas with moderate or high density of fast-food outlets [302]. On our walk we will surely encounter greater clustering of fast-food outlets and convenience stores around schools, as seen in numerous international studies [303,304,305,306,307,308]. We will find much higher brand name logo recognition in children in lower SES neighbourhoods [309]. Employment in “our neighborhood“ is more likely to be *within* fast-food or convenience store establishments—work that is also linked to greater obesity risk [310]. Healthy food and beverage options are less readily available [311,312,313] in this neighborhood. Instead, we are confronted with visual marketing—e.g., billboards, sidewalk signage, exterior-of-store ads, targeted screen media delivery—that strongly encourage us to uphold our unhealthy lifestyle choices such as sugar-added beverages, fast-food consumption and tobacco use [54,249,314,315]. This all means there are numerous additive interactions that explain socioeconomic inequalities in diet and obesity [316], and why the association between exposure to fast-food outlets and obesity is most pronounced for lower SES groups. But the stacked deck does not end here.

Remarkably, pharmacies (outlets that should be community beacons of medicinal products) might market cigarettes at relatively inexpensive prices [317]. Shelf-space and orientation within supermarkets and convenience stores in our neighborhood would likely be set up in such a way as to direct us to energy-dense, low nutrient foods [318,319,320,321,322]. Also inside, in-store marketing may direct us to a significant variety of (relatively) low-priced, high-sugar beverages [323]. On the other hand, healthier staples such as low-fat dairy products may be less available and more expensive [324].

The unhealthy foods in our neighborhood stores would be well-matched to targeted screen-media based marketing [325]. Media exposure can place children in front of celebrity endorsements for high-calorie, low-nutrient foods and beverages [326]. From a DOHaD perspective, the conversation concerning the built environment and drivers of behaviour is imperative; availability of convenience stores is associated with obesity in SES disadvantaged preschool children and higher presence of fast-food outlets with gestational diabetes [327,328].

More fast food outlets in SES disadvantaged communities can mean greater options to “super-size” high-fat foods and sugar-added beverages, obtain free prizes and with purchase, and the lure of branded mascots and licensed, recognizable characters [311]. Ages 3 to 5 are a critical time when taste and food preferences are being developed. Moreover, research shows that unhappiness and negative experiences during childhood are associated with adult food preferences toward comfort foods and away from healthier choices [329]. It is alarming, therefore, that in children within this young age group, knowledge of toys being offered by a multi-national purveyor of fast-food is significantly associated with higher frequency of eating at these locations [330]. Exposure to food marketing—including licensed and brand-equity characters (especially for children 3–12 years old)—is linked to significant, negative effects on food preferences, choices and consumption [331]. Consider that 50% of kids meals offered at North American fast-food and chain outlets exceed the WHO’s proposed daily (added) sugar recommendation of <5% of total energy [332].

Since the sum of combined marketing-dietary choice evidence meets Bradford Hill Criteria for causality in public health research (i.e., strength of association, dose-response, and consistent, biologically plausible and supported by experimental evidence), there is now clear substantiation for urgent restrictions on all forms of food marketing to children [333].

**Figure 4:**
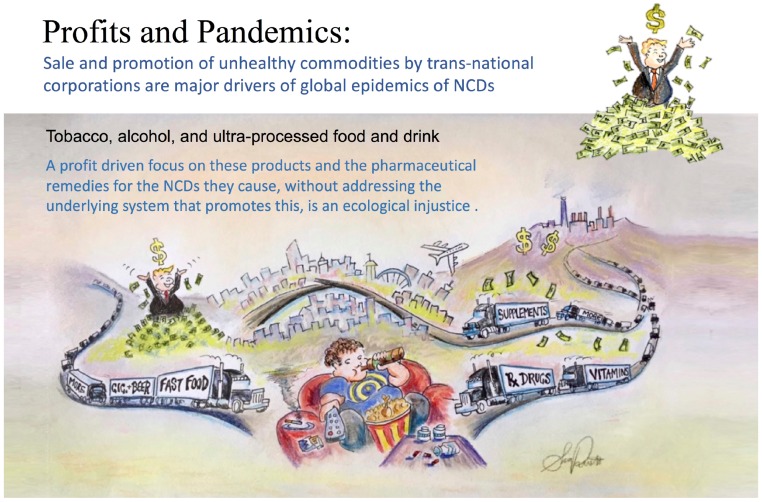
Transnational corporations profit from unhealthy commodities which directly contribute to health disparities in disadvantaged communities. These industries should have no role in the formation of health policy. There is no evidence that public-private partnerships are effective. Public regulation and market intervention are the only evidence-based measures (see [334]).

There is little dispute that transnational corporations *profit* from unhealthy commodities in our neighborhood. Profits—and the corporate political activity that maintains them—could easily drive taste-engineering decisions, and such decisions influence overconsumption of highly processed, calorie-dense, nutrient-poor foods [334,335,336] (Figure 4). The taste-engineering frame operates through three core pathways: environmental engineering (i.e., shaping the conditions in which food choices are made; e.g., point-of-purchase at check-out, all-you-can-eat buffets, vending machines in schools/hospitals, value meals etc.), cognitive engineering (i.e., marketing in all forms of media, sponsorship, celebrity etc.) and physiological engineering (i.e., exploitation of taste preferences for sodium, sugar, fat, caffeine) [337]. Put simply, taste-engineering is bathing the fetus, influencing the parents, the children and caregivers in our societies: this should be of the upmost importance in DOHaD discussions.

There is little doubt that effective marketing works. It shouldn’t be surprising that living in the vicinity of billboards advertising of highly processed foods is associated with decreased daily fruit or vegetable consumption [338]. Food advertising is especially effective in cajoling unhealthy food consumption when cognitive load is high. Thus, we have focused on delay discounting and cognitive resources in our perspective review to underscore the implications of its effects in our neighbourhood. Young adults from low SES backgrounds are more prone to advertising’s effects while under cognitive load [339].

In our neighbourhood, higher levels of persistent fatigue and psychological distress may enhance marketing messages in several key ways: First, as alluded to earlier, palatable, highly processed foods high in sugar, fat and sodium may induce temporary physiological changes to alleviate some degree of stress [76]. Similarly, tobacco and alcohol are also used as a means to mitigate stress [340]. Also, the appeal of readily available fast-food and ready-to-eat highly-processed foods could be magnified higher when fatigue, food insecurity, and economic pressures are at the forefront of thought processes [249]. Research involving pregnant (SES disadvantaged) women confirms that fatigue, irregular schedules, time pressure, and finances are intertwined with cravings for calorie-dense, nutrient poor foods [341]. In this way, omnipresent marketing messages and the subtle positioning of unhealthy foods within our neighbourhood are like a trap rigged toward the most vulnerable. Considering that experimental studies indicate a maternal ultra-processed westernized diet influences the offspring opioid system to reinforce preference for such foods [342], the time is past due for consideration of the neighbourhood as a womb, and for societal responsibility for the rigged, grey neighbourhood.

Thus, grey space is not solely about airborne particulate matter, soil contaminants, and environmental toxins in building materials. Rather, it is a completely disparate mental environment from that which green space represents. Grey space places a physiological and psychological, chemical and non-chemical, burden on available cognitive and other coping resources.

Finally, in regard to our emphasis on the importance of empathy in medical training, or lack thereof, in the context of neighborhood ecology: Research indicates that patients in our neighborhood may perceive the empathy of their physicians to be much lower than that afforded to physicians in affluent areas [343,344]. Moreover, in the relationship between physician empathy, humility and good medical outcomes, perception is likely a reality [345,346]. Given that inappropriate antibiotic prescriptions remain alarmingly high [347], it is worth noting that in the visualized neighborhood, one where primary care demands are typically strong, consultations shorter and *provider* stress is higher [348], a physician visit in our neighborhood is *more likely* to conclude with an antibiotic prescription [260,261,262]. Clearly, the “ecology“ of the patient visit and its direct and indirect effects on the microbiome requires further study.

## 20. Ecological Justice and the Erosion of Health

“*The tendency to disregard ecology in medical research may have far reaching consequences. For example, it facilitates the interpretation of the “environment” as “psychosocial environment”. The study of the environment is then implicitly relegated to psychology and social science. No wonder then that mental illness, in the orthodox view, gets a biological interpretation which skimps ecology.*”

van der Steen and Thung. In *Faces of Medicine: A Philosophical Study,* 1988 [349]

It is our contention that the term ecological justice (or ecological injustice) is well suited to capture the policies and practices that either promote or erode personal, community and planetary health. Healthy ecosystems involve interacting organisms operating within their abiotic (non-living, physical/chemical) environment. An ideal ecosystem (i.e., the “dwelling place” of each individual residence, their neighborhood and the collective city—socially, economically and environmentally supported) should allow each human to reach their potential, while at the same time ensuring planetary health.

The introduction of ecology into equity, justice and DOHaD discourse is a natural, if not necessary, development due to the microbiome revolution. The study of the microbiome has united researchers from virtually every branch of science and medicine. In the process, it has made salient the health relevancy of diverse ecosystems that operate from the largest scale (planetary) to the smallest (the microscopic dwellings on a single intestinal villus) [54].

Ecosystems—microbial and otherwise—can be manipulated by individual human behavior (lifestyle). However, the lifestyles of the host can be manipulated by forces within the larger “dwelling place” (e.g., neighborhood with more grey space and less green space; herein, high concentrations of fast-food outlets may be considered invasive species that limit growth and potential). Moving the lens even further out, the lifestyles (and policies and practices that maintain them) that promote environmental degradation, climate change and biodiversity loss are threats to ecological health. Since the effects of degradation and losses are (and will be in the future) shouldered by the disadvantaged [350], this is a matter of ecological justice. We urge that there is “No Health Without Ecological Health”.

Just as the DOHaD paradigm currently works diligently to prevent disease at its earliest point, so too it must identify the ways in which pro-social/environmental attitudes/behaviors and are shaped early in life. SES inequity is an obvious driver of health disparities, but to what extent does DOHaD place emphasis on research surrounding the societal cues—e.g., even miniscule hints of money, incidental exposure in experimental settings—that reinforce beliefs that socially advantaged groups should dominate socially disadvantaged groups [51]? This research suggests that improving the mental environment in disadvantaged communities—and thus, life-course health—requires education of the advantaged communities.

The crises of climate change, rapid urbanization, environmental degradation and gross biodiversity loss [351,352] in the context of a disconnection from nature [167,353], force emphasis from the DOHaD perspective that human health is dependent upon planetary health. DOHaD is making every effort to undo the NCD crisis at its fetal/first 1000 days origins; however, as a scientific/clinical movement it cannot promote *health* without widening the lens such that it visualizes the dysbiosis (life in distress) of Earthly ecosystems [236] and the reality that NCDs will be increasingly driven by climate change [354].

As much as natural environments have been associated with health promotion, research also demonstrates that “removal” or gross alteration of such environments is detrimental. Visible environmental degradation—whether through climate change, losses of millions of trees via the activity of invasive species, or human-generated industrial activity—has been clearly linked to diminished physical and mental health [355,356,357,358,359]. This degradation may have negative impacts on lifestyle behaviors such as leisure time spent outdoors in nature [360]. On the other hand, green remediation of vacated urban blight may be set up in such a way as to be aesthetically pleasing, yet also host biodiversity [361]. Relatively simple transformation of vacant urban lots with trees and vegetation has been associated with improved perceptions of the neighborhood, more physical activity, less stress and decreasing violence [362,363,364].

If we are to create change in ecological justice, if are to provide an equitable Petri dish for all humans to reach their potential, growing in an environment that acknowledges the interconnectivity and essentiality of biodiversity [365,366] to personal, public and planetary health, its emphasis through education, experience and the development of empathic concern in early life is imperative. For example, children who frequently experience nature are more likely to develop emotional affinity to and support for protecting biodiversity [367,368]. Outdoor childhood experience and engagement with nature has been reported to be a key driver of adult appreciation for the role of trees in human well-being [369,370].

**Figure 5:**
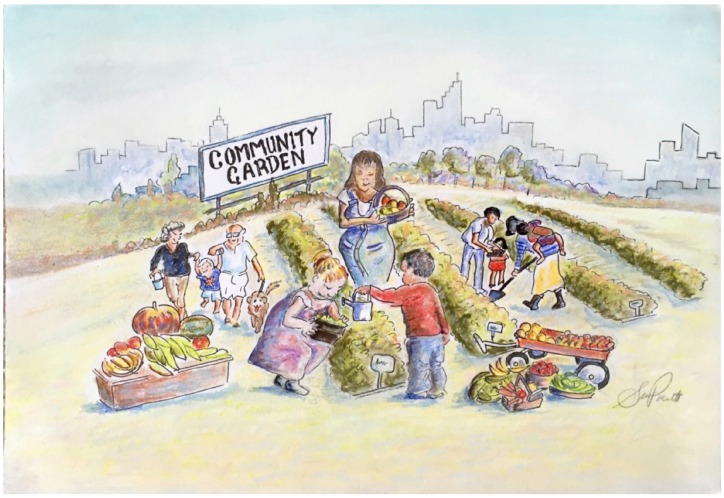
Community Gardens: Participation in personal, community and academic gardening programs has multiple social, personal and economic benefits. It increases nature-connectedness including the health benefits of microbial biodiversity, social and community cohesion, and positive emotions. It promotes a positive mental outlook and healthier behavior in general, including increased physical activity, increased intake of fruits and vegetables, and reduced fast-food consumption.

Participation in personal, community and academic gardening programs (Figure 5) may enhance intake and taste preference for fruits and vegetables [371,372], help maintain healthy BMI while diminishing fast-food consumption [373], increase positive emotions (e.g., joy, awe) and connectedness to nature [374]. Environmental education in elementary school early on in life can influence pro-environmental concerns. However, high levels of screen time have been associated with diminished concern for the environment [375]. Thus, we have emphasized screen time because it is no less a part of the DOHaD ecological picture, and it highlights the limitations of isolated, reductionist research in translation efforts.

Positive affect (vs. negative emotions/mood) which we have emphasized, is an important factor in encouraging prosocial behaviors [376,377] and appears to be critical in the development of pro-social behaviors in early life [378]. So too, environmentally sustainable behaviors and quality of life are significantly interrelated [379]. Engaging in pro-social activity, including in early life, encourages positive affect and reinforces further pro-social behavior [380,381,382]. This could be especially important when considering that in prospective research, associations between low pro-sociality at age 3 and subsequent behavioral problems are exacerbated in disadvantaged neighborhoods and low-performing schools [383].

## 21. Preparedness: Training Clinicians for Ecological Medicine

Despite what is already known concerning physical activity, nutrition and healthy weight management (and that best-practice guidelines for these variables are already in place), from the DOHaD perspective it should be alarming that so many women receive so little guidance from clinicians. For example, in a recent U.S.A. study, only about half of pregnant women received provider advice on these lifestyle factors [384]. Separate research from Australia also shows that few women receive clinical guidance on nutrition during pregnancy [385].

Volumes of emerging research in animal models demonstrate the importance of healthy dietary patterns, physical activity, adequate sleep and nutrition (and, for example, emergent microbiota-epigenetic mechanisms). These are informative and exciting from a DOHaD basic science perspective [11]. However, as Dubos warned in 1969 concerning technology research, there is a point at which enough is known about existing best practice where an emphasis should be placed on advocacy and barriers to change:

“*This is wonderfully entertaining, titillating kind of science fiction. We organize meetings about it in all sorts of pleasant places to talk about this, and that saves us the responsibility of walking across the street, where 100,000 children are being poisoned every day by lead in paint…something can be done immediately about this problem, but it is not being done because it is not of sufficient interest or as exciting intellectually*”.

[386]

Barriers to change include medical education. Despite recognizing the importance of exercise counseling in clinical settings, as many as 85% of graduating medical students in one North American study reported being to be ill-prepared to do so. The vast majority reported no training on patient interactions concerning exercise guidance [387]. Such findings shouldn’t be surprising given that less than half of all medical school curricula throughout the United States provide *any* formal training on physical activity [388]. Nutrition education also remains paltry—only 29% of medical schools in the USA provide the recommended minimum 25 h of nutrition education. Moreover, the bulk of nutrition instruction provided is still confined to preclinical (e.g., biochemistry) [389].

Again, from a DOHaD perspective, it should be alarming that medical school graduates entering pediatric residency are deficient in basic nutritional knowledge [390]. Fruit and vegetable intake among children in developed nations is notoriously low [391], especially in SES disadvantaged communities [392,393]. When as many as 87% of children from SES disadvantaged neighborhoods in North America consume *less* than two servings of fruits and vegetables daily [394], the clinical knowledge gaps should be of grave concern.

Baseline empathy among future doctors predicts resilience against typical declines in empathy toward patients during clinical training. This suggests that medical school admissions processes should consider this an asset, or at least something to be cultivated [395]. Declines in physician empathy are “a threat to health care quality“ [396], and this may be especially true in disadvantaged communities where, as we have already described, empathic concern by doctors is a critical part of outcomes. It may also help physicians understand the broad environmental crises and who will bear the force of climate change first, the disadvantaged.

It is commonly stated that physicians are often the most trusted sources about the environment, including matters of lifestyle and climate change as they pertain to health. But what is the basis of this privileged status when there is little physician training on neighborhood and planetary ecosystems in relation to chronic disease [397]? In recent surveys of American physicians specializing in allergy and lung function, only 7% to 10% reported being very knowledgeable about the association between climate change and health, with as many as 47% indicating that climate change is either not happening, it is mostly a natural phenomenon, or that human activity is no more a factor than natural processes [398,399,400]. These beliefs, and they are beliefs, are in direct opposition to the consensus of every legitimate science organization. The scales are well tipped toward humans as the dominant factor in climate changes over the last century and a half [401].

**Figure 6:**
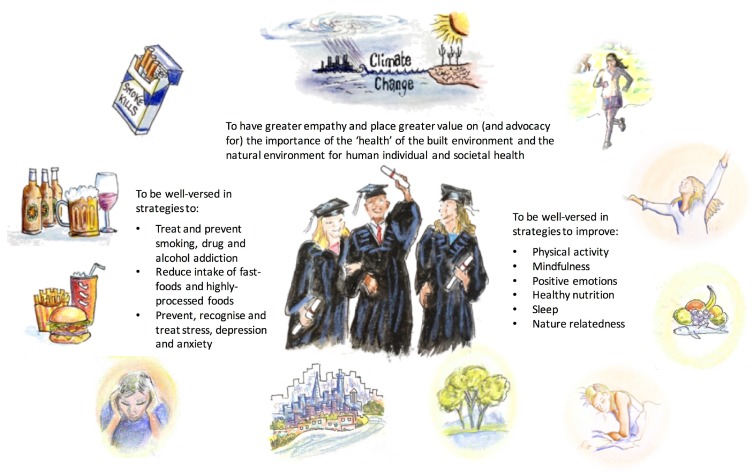
The future of human and societal health will depend on improved training and empathy of health care graduates, who need to be better prepared to understand the up-stream drivers, address the lifestyle diseases they need to prevent and treat, and to be advocates for solutions for the social, cultural and economic determinants of health.

Board exam/licensing questions to ensure knowledge accountability on ecological health (lifestyle and the influence of the environment), supervised clinical exposure and continuing medical education seems essential (Figure 6). Medical training devoid of course material, clinical exposure and exam accountability related to pharmaceuticals seems unimaginable. Medical training is one small, but important cog in the wheel of transforming the view of interconnectivity of life and lifestyle. The first step in this transformation will be undoing the ability of purveyors of ultra-processed foods from insinuating themselves in to medicine. At present, major health organizations accept tremendous sums of money from the largest players in the global soft drink industry; this, despite these companies lobbying against 97% of public health interventions intended to reduce junk food and improve nutrition [402].

## 22. Transforming Life: From Epigenetics to Advocacy

“*The study of man as an integrated unit, and of the ecosystems in which he functions, is grossly neglected…a very different kind of knowledge is needed to understand the nature of the cohesive forces which maintain man in an integrated state, physically, psychologically, and socially, and enable him to relate successfully to his environment.*”

Rene Dubos, 1965 [299]

It seems fair to ask whether or not the relegation of so-called “softer” science such as positive emotions, empathy, natural environments, marketing, biodiversity, nature-relatedness, pro-environmental and prosocial behavior etc. in the lower ranks of DOHaD discussions is in fact a collective professional act of delay discounting. Life-course *health* for the holobiont over the long-term will only be as good as these “soft” drivers of equity, cooperation, and societal decisions that determine the neighborhood and overall planetary health. In 1966 Dubos urged his fellow scientists to “*try, collectively, to imagine the world in which we want to live...improving the environment should not mean only correcting pollution or other evils of technological and urban growth*” [403].

Improving the environment in a holistic way will require imagination on the part of medical doctors, allied health professionals and scientists of all stripes. It will also require advocacy. The upstream drivers that bring increasing numbers of NCDs to the clinic are a product of a system of profits leading to pandemics [334], and absentia urban policies (covering quality of housing, employment opportunity, living wage compensation, social welfare) that that develop and/or maintain grey space. The policies that drive grey space and ecological inequalities, or at the very least, leave them in place, should be as unacceptable as a tobacco campaign aimed at children.

We implore all scientists, physicians and allied health professionals with an interest in DOHaD—especially with an eye toward social/environmental justice—to support advocacy in shifting the focus away from the well-trodden path of individual-only responsibility. Otherwise, we must surely ask to what extent headline-grabbing technological interventions (e.g., dietary supplements, probiotics that might address microbiota via epigenetics) can fulfill their expectations when environmental forces continue to erode the possibility of health, and drive dysbiosis by default? Dubos, perhaps said it more bluntly:

“*Developing counter technologies to correct new kinds of damage constantly being created by technological innovations is a policy of despair...we must try to imagine the kind of surroundings and of life we want, lest we end up with a jumble of technologies that will eventually smother body and soul.*”

[404]

## 23. Green Gentrification

It is not our contention that transformation of urban grey spaces into green spaces and blanket positive psychology interventions are ripe to proceed at an unbridled pace. Although available evidence provides plenty of generalized support for urban greening, what are the risks associated with transformation of urban neighborhoods—turning grey into green—such that they gentrify and displace the very people the action was intended to help [405,406]? As researchers learn more concerning how such transformations might take place (e.g., what constitutes best practice for natural environment quality, hypoallergenic greening, tree canopy height and density, design that might facilitate stress reduction or increase fear (hiding spots), aesthetic factors and more [407]), and the extent to which such changes have real-world benefits with worthwhile “effect sizes”, the unintended consequences of well-meaning transformations could be minimized by the active involvement of community stakeholders (including minorities, immigrants and others living with disadvantage) [408,409].

## 24. Conclusions

We have traversed broad-ranging topics that, at first glance, may seem only loosely connected to place-based characteristics and their relationships to to DOHaD. However, it is clear that the interconnectedness of urban life and lifestyle—biodiversity and bios, place and policy, practitioner and patient—can be illuminated by closer inspection of the microbiome zeitgeist. In particular, it demands discussion of the ways in which place-based health inequities are driven by upstream factors, such as marketing forces, neoliberal ideology and polices (of the absence thereof) that reinforce a dysbiosis on a gradient slanted toward the disadvantaged.

Available research allows us to draw straighter lines between the absence of healthy exposures and the presence of unhealthy exposures as they relate to the ecology of health—at the most minute and grand scales. We have attempted to underscore the unhealthy negative exposures that often escape DOHaD discourse; the connectivity, for example, between neighborhood fast-food clustering, the cognition of poverty itself, commercial advertising campaigns aimed at children, and how these might permeate the inner ecosystem in a place-based way. Thus, we have introduced the term ecological justice.

As we have attempted to make clear, ecological justice also involves equity in access to positive exposures in urban environments. Neighborhood access to natural environments and biodiversity are emerging as a means to narrow urban health inequities. Since positive psychological factors such as nature relatedness and optimism are emerging as health assets (the latter, optimism, enjoyed by the privileged at the neighborhood level), we need further understanding of ways in which place can determine allostatic load by way of its ability to foster or impede a healthy inner ecosystem.

In sum, a pivot in DOHaD research toward the upstream drivers of place-based *health* is required. Closer examination of parenting styles [410,411,412] in the urban grey space context, positive psychology interventions (also mindfulness, empathy, and relaxation response training) [413,414,415,416,417,418], and encouragement of time spent in natural environments via large-scale nature engagement campaigns [419,420] are worthy of study. We also need to learn more concerning emerging microbial applications in early life (e.g., through nature contact, controlled seeding of microbes and via environmental design) [421,422,423]. In other cases we simply need to learn more about effective delivery and advocacy. For example, how to promote engagement in uncomplicated exercise interventions such as walking (promotes positive affect even when participants expect the opposite) [424]. Now that a causal relationship between marketing and childhood food choices has been established [333], it is past-time to curb the unbridled marketing/sales of unhealthy foods/lifestyles to both children and adults in disadvantaged communities.

Transforming grey space, particularly its food environment, will require collective action; evidence indicates that it is insufficient to focus on exclusively on individual, interpersonal, organizational, and community levels [425]. Politics and policy will play a role, and this occurs in the context of cultural readiness for change—especially when interests, power and values of public and private actors are not in alignment [426]. However, it seems clear that simple rinse-and-repeat messages to eat healthy via pyramids and plates are far from effective. Rather, emerging research shows that when the topic of healthy nutrition and the food environment is presented to youth from as a food justice and power inequity issue, interest is piqued [427]. This may be especially true in youth from SES disadvantaged communities. Informed on the relationship between food environment issues and poor dietary behavior, empowered youth became community advocates for change, educating parents, teachers and others [428]. However, this same research shows that community efforts must be supported at the larger societal, policy level.

Early indications from Mexico’s innovative tax on nonessential energy-dense foods show a decline in purchases of such items, particularly in lower SES households [429]. Municipal efforts, such as the policy to eliminate sugar-sweetened beverage sales on Boston city property, has also reduced sugar and caloric intake, with potential community-wide benefits [430]. Transformation of vending machine contents in urban parks has also been shown to bring caloric and sugar content in alignment with guidelines for children [431]. Encouragingly, research efforts aimed at evaluating the combined influences of early-life healthy eating, physical activity and nature relatedness in urban children are underway [432].

Transformation of grey space and equity in maximizing the odds for the realization of health—personal, societal and planetary—may occur more rapidly when rights are kept in the forefront of our minds. As we stated at the outset, the United Nations and other international groups state quite clearly that children have the right to thrive, and that all humans have a fundamental right to live in an environment that supports their overall health and well-being [433]. Often this is interpreted almost exclusively as an environment that is absent chemical toxins [434]. Here, we have used a wide-angle lens in how we interpret the “environment“, and its relationship to human rights to health.

We underscore that a mental environment that is absent of green space and filled with “grey” visual, auditory and total sensory cajoling toward unhealthy lifestyle behaviours is *at odds* with this fundamental right to thrive. It is most certainly at odds with the right of children to develop a healthy respect for the natural environment. Our concern is that the connections between other societal-induced physical contaminants within built landscapes (the exposures to which can be manipulated by marketing) and trans-generational health routinely evade discussion. We need to pull the curtain back and continue to show our youth how they and their neighbourhoods, and indeed even the health organizations they are groomed to trust, can be manipulated by marketers of dysbiosis [402].

We agree with others who have pointed out through research applications that in matters of SES disadvantage, policy and the environment, there often remains more plasticized rhetoric than forthright action [435]. DOHaD research and advocacy can help speed up rhetoric to translation. In keeping with our theme of positive emotions and optimism, and honouring the dedication of the youth who helped transform two corner stores in a disadvantaged Los Angeles community [428], reaching out in multiple directions to individuals, shopkeepers and society as they attempted to brighten a touch of grey, we close on some of the last words ever written by Rene Dubos:

“*I am eighty years old as I write these lines…I am still vigorous enough not only to resent many aspects of modern civilization but more importantly to enjoy the world and have faith in its future...I have become convinced that resiliency is a universal attribute of all living organisms—from natural ecosystems to individual human beings; it is also one of the most important. In living organisms, resiliency implies the ability both to recover from traumatic experiences and to create new values during the very process of recovery*”.

[436]
